# Adaptive Behaviour and Cognitive Skills: Stability and Change from 7 Months to 7 Years in Siblings at High Familial Risk of Autism Spectrum Disorder

**DOI:** 10.1007/s10803-018-3554-5

**Published:** 2018-04-03

**Authors:** Erica Salomone, Elizabeth Shephard, Bosiljka Milosavljevic, Mark H. Johnson, Tony Charman, The BASIS Team, Simon Baron-Cohen, Rachael Bedford, Patrick Bolton, Susie Chandler, Mayada Elsabbagh, Janice Fernandes, Holly Garwood, Teodora Gliga, Kristelle Hudry, Emily J. H. Jones, Greg Pasco, Andrew Pickles, Leslie Tucker, Agnes Volein

**Affiliations:** 1 Department of Psychology, University of Turin, Via Po, 14, 10123 Turin, Italy; 2 Department of Psychology, Institute of Psychiatry, Psychology & Neuroscience, King’s College London, London, UK; 3 Centre for Brain and Cognitive Development, Birkbeck College, University of London, London, UK; 4 Department of Psychology, University of Cambridge, Cambridge, UK

**Keywords:** Cognitive ability, Adaptive behaviour, High familial risk, Prospective study

## Abstract

Cognitive and adaptive behaviour abilities early in life provide important clinical prognostic information. We examined stability of such skills in children at high familial risk for ASD who either met diagnostic criteria for ASD at age 7 years (HR-ASD, *n* = 15) or did not (HR-non-ASD, *n* = 24) and low-risk control children (LR, *n* = 37), prospectively studied from infancy. For both HR groups, cognitive skills were consistently lower across time than those of LR children. HR-ASD children showed increasing difficulties in adaptive behaviour over time compared to LR children, while the HR-non-ASD children showed no such difficulties. This pattern of change may inform our understanding of developmental profiles of HR siblings beyond core ASD symptoms.

The diagnostic criteria for autism spectrum disorder (ASD) in DSM-5 (APA 2013) introduce the notion that symptomatology “may not become fully manifest until social demands exceed limited capacities” or, conversely, that it “may be masked by learned strategies in later life”. This novel approach stresses the importance of assessing the person/environment relationship, which can vary over time and across individuals, when evaluating the presence and impact of core symptoms of ASD. The assessment of adaptive behaviour, that is the individual’s ability to translate his/her cognitive ‘capacity’ into real-life ‘competencies’ (Sparrow and Cicchetti 1985), complements this framework. Given the growing recognition of the variability of developmental trajectories in the adaptive behaviour domain (Szatmari et al. 2015), irrespective of ASD core symptomatology, particularly in higher-functioning individuals (Klin et al. 2007; Saulnier and Klin 2007; Kanne et al. 2011) the evaluation of adaptive behaviour as part of long-term outcomes is now thought to be of critical importance (Magiati et al. 2014).

Since adaptive behaviour is inherently understood as an age-dependent construct, prospective studies within a familial high-risk (HR) design provide a unique opportunity not only to capture the emergence of impairment over time in siblings who eventually meet diagnostic criteria for ASD, but also to identify difficulties—or, conversely, strengths—in those who do not. Data on the emergence of group differences over time in cognitive skills are available from longitudinal ‘at-risk’ design studies (Landa et al. 2012; Messinger et al. 2013), however fewer data are available on adaptive behaviour and the measures for adaptive behaviour, usually the Vineland II (Vineland Adaptive Behaviour Scales, VABS, Sparrow et al. 2005), are generally only listed as a contributor to the diagnostic process or decision (Jones et al. 2014). HR children have been reported to show lower adaptive behaviour at 20 months (Toth et al. 2007), 36 months (Zwaigenbaum et al. 2012), and in mid-childhood (Shephard et al. 2017), but stability across development has not yet been investigated in HR sibling studies, with the exception of narrowly focused analyses of communication and gross motor impairments up to 36 months of age (Hudry et al. 2014; Leonard et al. 2014).

In respect to cognitive skills, there is evidence of differences between low risk (LR) controls and HR-non-ASD siblings, although a comparison across studies is made difficult due to the differing clinical outcomes categorizations used (e.g., atypically developing LR children are sometimes but not always excluded from the analyses), and the fact that for some studies cognitive measures contributed to the outcome classification. In infancy, HR and LR siblings do not differ in cognitive skills at 6 months (Ozonoff et al. 2014). However, as early as at 12 months, HR siblings classified with atypical development (HR-non-ASD children, defined as having signs of developmental delay and/or subclinical ASD symptoms) score lower on cognitive assessments than typically developing LR siblings (Macari et al. 2012; Ozonoff et al. 2014), and the difference persists at 36 months (Landa et al. 2012; Messinger et al. 2013; Ozonoff et al. 2014). At school-age, both Warren et al. (2012) and Miller et al. (2015) reported only non-significant trends for lower scores on non-verbal cognitive measures in HR siblings. Gamliel et al. (2009) investigated cognitive developmental trajectories over time (4–54 months) in HR-ASD, HR-non-ASD and LR siblings, reporting no significant differences in cognitive scores over time and a similar pattern of change across groups. In contrast, Ozonoff et al. (2014) found different developmental trajectories, with the LR typically developing children sharply increasing on all scales with age, a slower growth on verbal scales for the HR typically developing children, significantly lower rate of change for ASD children, and an intermediate performance for the atypically developing HR-non-ASD children with a slower rate of growth that amplified from 12 to 36 months.

In summary, there is conflicting evidence of group differences and stability of cognitive skills in HR sibling studies, and the limited amount data on adaptive behaviour hinders our understanding of the relationship between these two constructs, both on a theoretical and clinical level. In the current study we report on the stability and change of both cognitive and adaptive behaviour skills, for the first time between infancy and mid-childhood, in a prospective study of LR and HR children. Study participants, prospectively followed-up from the first year or life to age 7, included both those who met diagnostic criteria for ASD at age 7 years (HR-ASD) and those who did not (HR-non-ASD).

Based on the available evidence on the emerging ‘broader autism phenotype’ (BAP) in HR-non-ASD siblings (Messinger et al. 2013; Charman et al. 2017) and the previous reports on progressively worsening or stably low trajectories of adaptive behaviour in the majority of children with ASD (Flanagan et al. 2015; Szatmari et al. 2015), we predicted the following pattern of findings:

1. Across all time points, cognitive and adaptive functioning would be lower in the HR-ASD group than in the LR and HR-non-ASD groups, and lower in the HR-non-ASD group than in the LR group.
2. Group differences in cognitive functioning would be stable across time, but the gap in adaptive functioning between the HR-ASD and both LR and HR-non-ASD groups would widen with time.

## Methods

### Participants

Participants (*n* = 104; 54 HR, 50 LR) were recruited from the British Autism Study of Infant Siblings (BASIS; http://www.basisnetwork.org) network database. At enrolment, all HR children (21 males, 33 females) had a sibling or half-sibling (hereafter probands) with a community clinical ASD diagnosis confirmed by two expert clinicians in the research team using parent-report of symptoms from the Development and Wellbeing Assessment (DAWBA; Goodman et al. 2000) and the Social Communication Questionnaire (SCQ; Rutter et al. 2003). None of the children, probands or extended family members were reported to have any conditions related to ASD (such as fragile X syndrome, tuberous sclerosis). The LR control infants (21 males, 29 females) with no family history of ASD and a typically developing older sibling (scoring below the SCQ cutoff) were recruited from the Birkbeck Centre for Brain and Cognitive Development research volunteer database. Participants were invited to complete research visits at 7, 14, 24, 36 and 84 months; the 84-month visit was attended by 42 (78%) HR siblings and 37 (74%) LR controls. The retained sample did not differ from the non-retained sample in 36-month levels of ASD symptomatology (on the Autism Diagnostic Observation Schedule, Second Edition, ADOS-2; Lord et al. 2012; the Social Responsiveness Scale, Second Edition, SRS-2; Constantino and Gruber 2012 or the SCQ; Rutter et al. 2003), developmental level (on the Mullen Scales of Early Learning; MSEL, Mullen 1995), adaptive behaviour (on the Vineland Adaptive Behavior Scales, Second Edition, Vineland-II; Sparrow et al. 2005), or family income (all *p*s > .4). At the 84-month follow-up a diagnostic outcome (ASD, non-ASD; Shephard et al. 2017) was assigned. Three experienced researchers and the lead clinician reviewed all available clinical information on ASD symptomatology and functioning, including from the Autism Diagnostic Interview-Revised (ADI-R, Le Couteur et al. 2003) and the ADOS-2 (Lord et al. 2012) and as a team assigned clinical consensus best estimate diagnosis of ASD according to DSM-5 criteria (APA 2013). The diagnostic process at the 84-month visit included review of all clinical information previously obtained, including from the diagnostic decision at the 36-month visit, but was ultimately made as to whether the child *currently* met DSM-5 criteria for ASD. None of the LR children met DSM-5 criteria for ASD or had a community clinical ASD diagnosis. In the HR group, 15 met diagnostic criteria for ASD (‘HR-ASD’ group) and 24 did not (‘HR-non-ASD’ group); three children who had previously been diagnosed with ASD at 36 months did not retain the diagnosis at 84 months (‘lost diagnosis’ group) and, following our previous report of clinical outcomes (Shephard et al. 2017), were excluded from the analysis. Overall household income reported across visits and educational level at the last visit was significantly higher in LR than in HR families (*p* = .003 and *p* < .001). Ethical approval was obtained from the NHS National Research Ethics Service (NHS RES London REC 08/H0718/76; 14/LO/0170). Parents provided written informed consent and children, wherever possible given developmental level, provided written informed assent.

## Measures

### Cognitive Skills

At 7, 14, 24 and 36 months the MSEL (Mullen 1995) was used to assess cognitive skills. At 84 months, children completed the Wechsler Abbreviated Scale of Intelligence—Second Edition (WASI-II, Wechsler 2011). Standardised, age-normed quotients (mean 100; SD 15) for the Mullen Early Learning Composite (ELC) and the WASI full-scale IQ (FSIQ) were used in analyses of Full Scale IQ (FSIQ). Mullen non-verbal and verbal IQ scores (Mullen NVIQ and VIQ) were obtained averaging T scores (mean 50; SD 10) of subscales (for the NVIQ: gross motor, fine motor, visual reception; for the VIQ: receptive language, expressive language) and subsequently transforming the T scores into deviation IQ scores (mean 100; SD 15). The Mullen NVIQ and VIQ scores were used with the WASI Perceptual Reasoning Index (PRI) and Verbal Comprehension Index (VCI) quotients (mean 100; SD 15) in the analysis of, respectively, non-verbal and verbal skills. The MSEL and WASI are not equivalent but both provide a measure of verbal, non-verbal and overall cognitive functioning and have been used to assess change in cognitive skills in children with ASD from toddlerhood to mid-childhood (Clark et al. 2017). In this sample the correlation between the Mullen ELC at 36 months and the WASI FSIQ at 84 months was moderate across groups (Pearson’s R = .424, *p* < .001).

### Adaptive Behaviour Skills

At all research visits parents completed the Vineland-II (VABS, Sparrow et al. 2005), rating their child’s current level of functioning across the domains of communication, daily living and socialization. Age-normed Standard Scores (mean 100; SD 15) for each domain and the adaptive behavior composite (ABC) were used in analyses.

### Data Analysis

To explore group differences and stability of IQ and adaptive behaviour over time, we carried out mixed-model ANOVAs with time (7, 14, 24, 36, and 84-month visits) as the within-subjects variable and ASD outcome group at 84 months (LR, HR-non-ASD, HR-ASD) as the between-subjects factor for FSIQ (Mullen ELC SS and WASI FSIQ) and adaptive behaviour skills (Vineland II Adaptive Behaviour Composite and Socialization, Communication and Daily Living Skills domains). Similarly, to explore differences and stability of non-verbal and verbal cognitive skills over time, we conducted mixed-model ANOVAs for NVIQ scores (obtained from Mullen NVIQ and WASI PRI) and VIQ scores (obtained from Mullen VIQ and WASI VCI) with outcome group as the between-subjects variable and time as the within-subjects variable. When Mauchly’s sphericity test was violated, degrees of freedom were corrected using Greenhouse–Geisser estimates. Bonferroni-corrected pairwise comparisons were conducted to follow-up on significant main effects of group and time. Significant group × time interactions were investigated further by evaluating the simple main effects of group separately for each time point and of time separately by group (Tables 1 and 2).

## Results

**Fig. 1:**
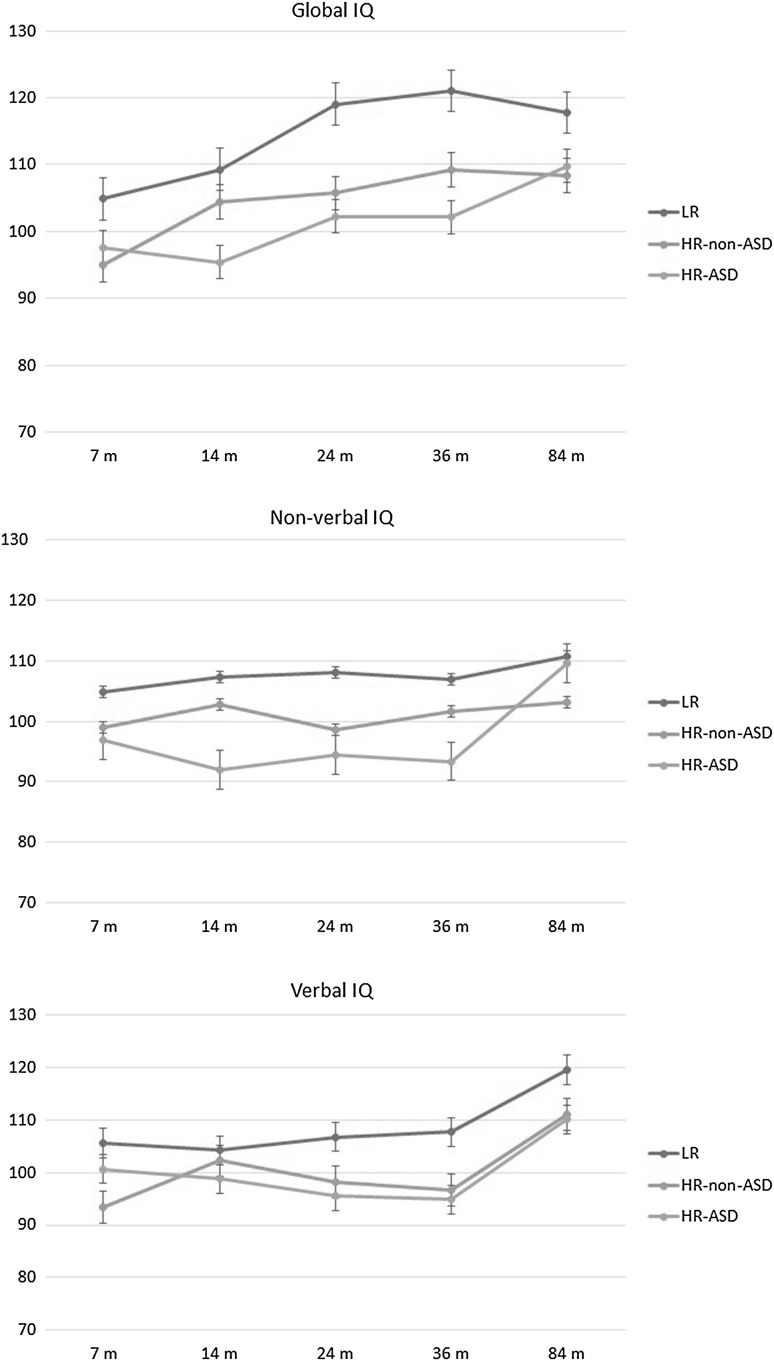
Change in cognitive scores over time. Standard errors of the mean are represented in the figure by the error bars attached to each line

**Fig. 2:**
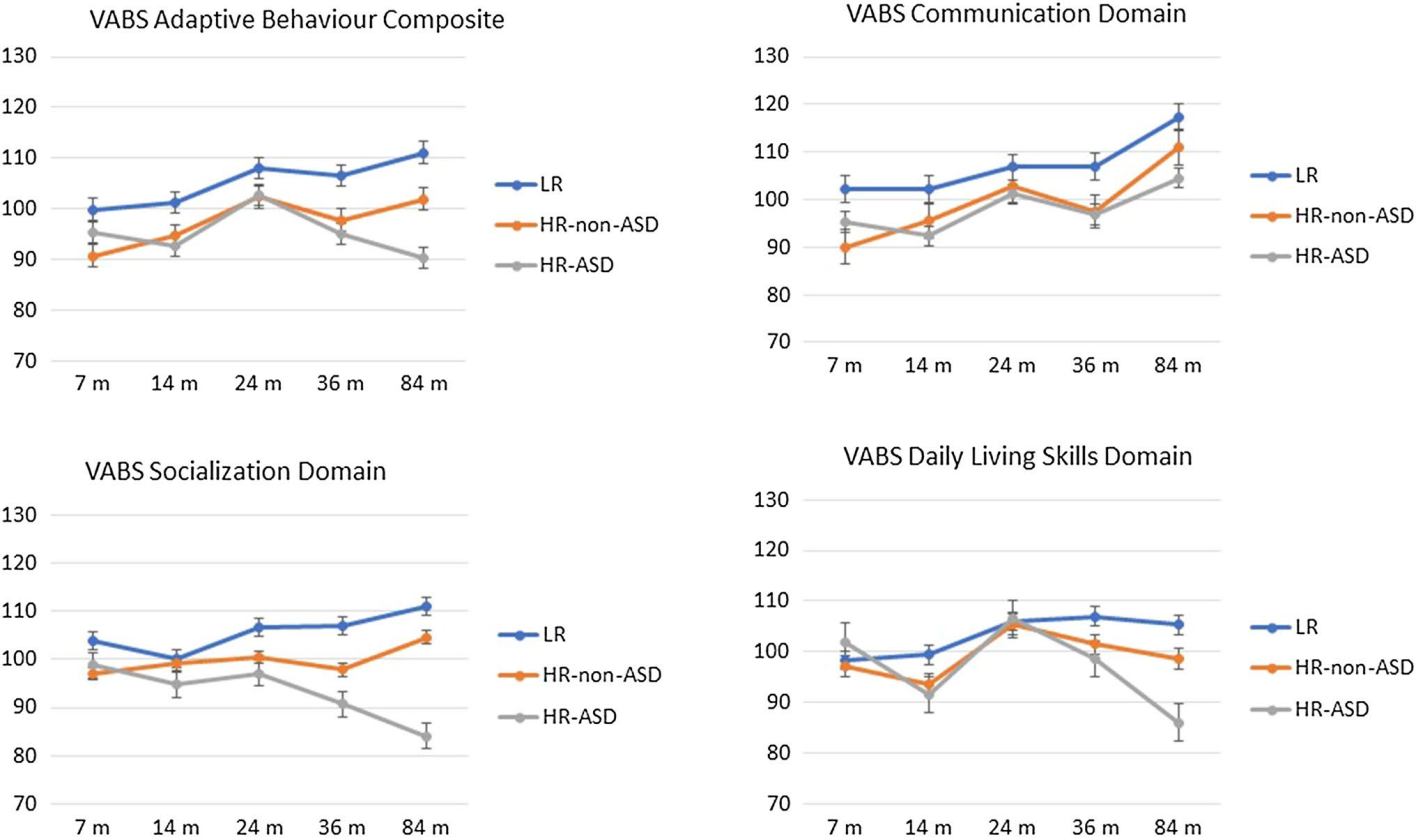
Change in adaptive behaviour scores over time. Standard errors of the mean are represented in the figure by the error bars attached to each line

Group means for cognitive skills and adaptive functioning are presented by time point in Table 1 and Figs. 1 and 2. *F*(df) values, *p* values and effect sizes are reported in Table 2.

**Table 1:** Cognitive and adaptive behaviour skills at 7, 14, 24, 36 and 84 months

	7-month visit			14-month visit			24-month visit			36-month visit			84-month visit		
	LR	HR-non-ASD	HR-ASD	LR	HR-non-ASD	HR-ASD	LR	HR-non-ASD	HR-ASD	LR	HR-non-ASD	HR-ASD	LR	HR-non-ASD	HR-ASD
FSIQ															
M	104.87	95	97.64	109.3	104.48	95.43	119.07	105.74	102.29	121.07	109.26	102.14	117.77	108.35	109.79
SD	10.62	9.2	16.82	15.87	11.74	14.65	13.46	16.02	18.59	13.03	17.05	24.8	12.14	12.9	21.36
n	30	23	14	30	23	14	30	23	14	30	23	14	30	23	14
NVIQ															
M	104.93	98.92	96.81	107.31	102.81	91.93	108.14	98.66	94.48	106.99	101.7	93.36	110.73	103.13	109.57
SD	13.83	12.93	19.19	12.32	10.05	13.49	12.62	11.7	16.13	10.32	13.98	16.5	12.7	9.97	18.26
n	30	23	14	30	23	14	30	23	14	30	23	14	30	23	14
VIQ															
M	105.63	93.3	100.69	104.24	102.23	98.74	106.74	98.24	95.54	107.69	96.71	94.86	119.58	111.09	110.14
SD	13.22	9.95	18.72	13.82	12.53	14.53	10.46	12.76	14.78	10.29	11.89	20.3	14.23	15.22	25.87
n	33	23	14	33	23	14	33	23	14	33	23	14	33	23	14
ABC															
M	99.9	90.73	95.27	101.34	94.68	92.8	108.03	102.32	102.6	**106.48**	**97.82**	**95.07**	**111.1**	**101.86**	**90.27**
SD	13.83	16.76	13.77	9.44	14.03	13.35	12.92	8.32	12.37	**9.19** ^**a**^	**9.83** ^**b**^	**16.65** ^**b**^	**6.97a**	**12.85b**	**15.46c**
n	29	22	15	29	22	15	29	22	15	29	22	15	29	22	15
Comm															
M	102.13	90.04	95.33	102.10	95.48	92.33	106.77	102.83	101.13	107.03	97.48	96.87	117.43	110.87	104.53
SD	14.85	22.55	12.54	7.75	17.28	16.22	12.26	8.78	14.21	11.07	9.04	19.33	10.87	13.94	18.08
n	30	23	15	30	23	15	30	23	15	30	23	15	30	23	15
Soc															
M	103.87	97.05	98.73	100.1	99.05	94.73	**106.63**	**100.41**	**97.13**	**106.9**	**97.82**	**90.67**	**110.9**	**104.59**	**84**
SD	12.97	16.47	16.44	10.9	13.65	15.69	**13.84** ^**a**^	**7.45** ^**ab**^	**10.32** ^**b**^	**9.87** ^**a**^	**12.05** ^**b**^	**15.92** ^**b**^	**5.29** ^**a**^	**11.89** ^**a**^	**17.49** ^**b**^
n	30	22	15	30	22	15	30	22	15	30	22	15	30	22	15
DLS															
M	98.24	97.09	101.93	99.38	93.7	91.53	106	105.48	106.47	107.03	101.48	98.67	**105.31**	**98.7**	**86.07**
SD	16.75	18.04	15.03	10.07	12.76	13.8	10.37	9.43	11.67	8.05	9.03	18.79	**9.29** ^**a**^	**10.36** ^**a**^	**15.65** ^**b**^
n	29	23	15	29	23	15	29	23	15	29	23	15	29	23	15

FSIQ (Full scale IQ): Mullen Early Learning Composite Standard Score and Wechsler Abbreviated Scale of Intelligence (WASI) Full Scale IQ; NVIQ (Non-verbal IQ): Mullen gross motor, fine motor, visual reception and WASI Perceptual Reasoning Index; VIQ (Verbal IQ): Mullen Expressive and Receptive Language and WASI Verbal Comprehension Index; ABC, Comm, Soc and DLS = Vineland Adaptive Behaviour Composite Standard Score, Communication, Socialisation and daily living skills domains Standard Score

Simple main effects of significant group × time interactions (*p* < .05) are marked in bold. Groups marked with different superscript letters (a, b, c) differed significantly with Bonferroni correction applied (*p* < .05)

**Table 2:** Stability of cognitive and adaptive behaviour skills by ASD outcome at 84 months

	ANOVAs		
	Group effects	Time effects	Group × time effects
*FSIQ*	*F*(2, 64) = 9.62, *p* < .001, $\eta _{P}^{2}$ = .231	*F*(4, 256) = 13.09, *p* < .001, $\eta _{P}^{2}$ = .170	ns
*NVIQ*	*F*(2, 64) = 8.99, *p* < .001, $\eta _{P}^{2}$ = .219	*F*(3.47, 222.38) = 4.77, *p* = .002, $\eta _{P}^{2}$ = .069*	ns
*VIQ*	*F*(2, 67) = 6.98, *p* = .002, $\eta _{P}^{2}$ = .172	*F*(2.84,189.98) = 16.49, *p* < .001, $\eta _{P}^{2}$ = .197*	ns
*ABC*	*F*(2, 63) = 8.92, *p* < .001, $\eta _{P}^{2}$ = .221	*F*(3.48, 219.12) = 8.44, *p* < .001, $\eta _{P}^{2}$ = .118*	*F*(6.96, 219.12) = 2.47, *p* = .019, $\eta _{P}^{2}$ = .073*
*Comm*	*F*(2, 65) = 6.28, *p* = .003, $\eta _{P}^{2}$ = .162	*F*(3.11, 201.88) = 18.31, *p* < .001, $\eta _{P}^{2}$ = .220*	ns
*Soc*	*F*(2, 64) = 11.32, *p* < .001, $\eta _{P}^{2}$ = .261	ns	*F*(8, 256) = 4.59, *p* < .001, $\eta _{P}^{2}$ = .125
*DLS*	*F*(2, 64) = 3.40, *p* = .039, $\eta _{P}^{2}$ = .096	*F*(2.83, 181) = 10.38, *p* < .001, $\eta _{P}^{2}$ = .140*	*F*(5.66, 181) = 3.43, *p* = .004, $\eta _{P}^{2}$ = .097*

FSIQ (Full scale IQ): Mullen Early Learning Composite Standard Score and Wechsler Abbreviated Scale of Intelligence (WASI) Full Scale IQ; NVIQ (Non-verbal IQ): Mullen gross motor, fine motor, visual reception and WASI Perceptual Reasoning Index; VIQ (Verbal IQ): Mullen Expressive and Receptive Language and WASI Verbal Comprehension Index; ABC, Comm, Soc and DLS = Vineland Adaptive Behaviour Composite Standard Score, Communication, Socialisation and daily living skills domains Standard Score

*Mauchly’s sphericity test violated: degrees of freedom corrected using Greenhouse–Geisser estimates

### Cognitive Skills

#### Global IQ

There was a significant main effect of group. FSIQ (Mullen ELC / WASI IQ) was significantly higher in LR children than in both HR-non-ASD (*p* = .003) and HR-ASD (*p* = .001). There was also a significant main effect of time. FSIQ was stable from 7 to 14 months but significantly higher at 24, 36 and 84 months than both at 7 months (all *p*s < .001) and 14 months (*p*s ranging from .007 to .023). The group × time interaction was not significant.

#### Non-Verbal IQ

There was a significant main effect of group. NVIQ (Mullen NVIQ / WASI PRI) was significantly higher in LR children than in both HR-non-ASD (*p* = .015) and HR-ASD (*p* = .001). There was also a significant main effect of time. NVIQ was significantly higher at 84 months than at 7 months (*p* = .045), 14 months (*p* = .025), 24 months (*p* = .007) and 36 months (*p* = .003). The group × time interaction was not significant.

#### Verbal IQ

There was a significant main effect of group. VIQ (Mullen VIQ/WASI VCI) was significantly higher in LR children than in both HR-non-ASD (*p* = .005) and HR-ASD (*p* = .017). There was also a significant main effect of time. VIQ was significantly higher at 84 months than at all previous timepoints (all *p*s < .001). The group × time interaction was not significant.

### Adaptive Behaviour Skills

#### VABS Adaptive Behavior Composite

There was a significant main effect of group. VABS ABC scores were lower in both HR-ASD and HR-non-ASD than in LR children (respectively, *p* = .001 and *p* = .005). There was also a significant main effect of time. VABS ABC at 24 months was significantly higher than at 7 and 14 months (both *p*s < .001). The interaction term group × time was significant. At 36 months, VABS ABC scores were lower in both HR-ASD and HR-non-ASD children than in LR children (respectively, *p* = .008 and *p* = .029), but the two HR groups did not differ from each other. At 84 months, the three groups were all different from each other, with HR-ASD children scoring lower than both LR and HR-non-ASD children (*p* < .001 and *p* = .010) and HR-non-ASD children scoring lower than LR children (*p* = .017). In LR children, VABS ABC scores at 7 and 14 months were lower than at 84 months (*p* = .008 and *p* = .005). In HR-non-ASD children, VABS ABC at 7 months was lower than at 24 (*p* = .011) and 84 months (*p* = .033). In HR-ASD children, VABS ABC increased from 14 to 24 months (*p* = .034), but at 84 months was lower than at 24 months (*p* = .009).

#### VABS Communication Domain

There was a significant main effect of group. VABS Communication scores were significantly higher in LR children than in both HR-non-ASD (*p* = .015) and HR-ASD (*p* = .012) children. There was also a significant main effect of time. VABS Communication skills were similar at 7 and 14 months, increased at 24 months (*p* = .013), remained stable at 36 months and increased significantly from 36 to 84 months (*p* < .001). The group × time interaction was not significant.

#### VABS Socialization Domain

There was a significant main effect of group. Both HR-ASD and HR-non-ASD children had lower VABS Socialization skills than LR children (respectively, *p* < .001 and *p* = .048), but the two HR groups were not different from each other. The main effect of time was not significant. There was a significant interaction of group × time. At 24 months, VABS Socialization skills were lower in HR-ASD children than in LR children (*p* = .030). At 36 months, both HR-ASD and HR-non-ASD children had lower skills than LR children (respectively, *p* < .001 and *p* = .029). At 84 months, HR-ASD children had lower skills than both LR and HR-non-ASD children (both *p*s < .001). In LR children, VABS Socialization skills were significantly higher at 84 than at 14 months (*p* = .006). In HR-non-ASD children, skills remained stable over time. In HR-ASD children, VABS Socialization skills at 84 months were significantly lower than at 7 and 24 months (*p* = .006 and  .008).

#### VABS Daily Living Skills Domain

There was a significant main effect of group. VABS Daily Living Skills (DLS) were lower in HR-ASD children than in LR children (*p* = .049). There was also a significant main effect of time. At 7 months VABS DLS were lower than at 24 months. At 14 months, VABS DLS were lower than at 24 months (*p* < .001) and 36 months (*p* = .001), but not different than at 84 months. Both at 24 and 36 months VABS DLS were higher than at 84 months (respectively, *p* < .001 and .004). There was a significant group × time interaction, with VABS DLS in HR-ASD children at 84 months lower than both those of LR children (*p* < .001) and HR-non-ASD children (*p* = .004). In both LR and HR-non-ASD children, VABS DLS increased from 14 to 24 months (*p* = .020 and *p* < .001), then remained stable. In HR-ASD children, VABS DLS increased from 14 to 24 months (*p* < .001), remained stable from 24 to 36 months and by 84 months were lower than both at 24 and 36 months (*p* < .001 and .001).

## Discussion

We investigated the development of cognitive and adaptive behaviour in children at high and low familial risk for ASD by diagnostic outcome at age 7 years. We firstly hypothesised group differences in these skills, with HR-ASD children showing the poorest cognitive and adaptive behaviour skills and the HR-non-ASD group in an intermediate position between HR-ASD and LR children. However, we did not find evidence of a gradient of competences from LR to HR-non-ASD to HR-ASD children and found that both HR groups displayed lower cognitive and adaptive skills compared to LR children across time points. Across groups, both cognitive and adaptive skills increased over time, with lower scores at 7 and 14 months than at later time-points, followed by stable scores from 24 months, reflecting the lower stability of skills in infancy. The analysis of non-verbal and verbal cognitive skills separately also showed lower skills in both HR groups compared to the LR children and, across groups, higher skills at 84 months than at earlier timepoints.

Secondly, we hypothesised that the gap between HR-ASD children and both LR and HR-non-ASD children would widen with time for adaptive skills but not for cognitive skills. We found that the gap between LR children and both HR groups remained stable over time for cognitive skills but widened progressively over time for adaptive behaviour. In our sample of cognitively able children, findings align with the robust evidence of a higher discrepancy between cognitive and adaptive skills in high-functioning individuals (Carpentieri and Morgan 1996; Liss et al. 2001; Charman et al. 2011). In our sample, cognitive skills were consistently lower for both HR-non-ASD and HR-ASD children than LR children. In contrast, we found an increasing gap in adaptive behaviour between HR and LR children from 3 years onwards, with the HR-ASD children showing poorer skills than both the LR and the HR-ASD children at the final time of assessment (7 years). Further, we found distinct patterns of discrepancies by adaptive behaviour domain. Communication skills remained stably lower over time for both HR groups compared to LR children. HR-non-ASD children had lower socialization skills than LR children at 3 years, but at 7 years they did not differ from LR children in either socialization or daily living skills. Conversely, HR-ASD children showed a progressive decrease in socialization skills from age 2, and daily living skills were significantly lower at age 7 compared to both LR and HR-non-ASD children. The age-related decline in daily living skills for the HR-ASD group is consistent with well-established findings of a negative correlation between adaptive behaviour skills and chronological age in children with ASD (Liss et al. 2001; Perry et al. 2009), which reflects the challenges of the out-of-home environments where children are expected to spend more time as they grow. In fact, while increased exposure to those social contexts at school-age may promote the development of socialization skills in typical development (as reflected in the improvement at age 7 reported for HR-non-ASD children), the effect of such social challenges, when not buffered by specific psycho-educational interventions, may be detrimental for children with ASD. In our study, parents completed a minimal survey of access to special education plans at school and use of intensive ASD specific interventions between the ages of 3 and 7 years, indicating, in line with the expected service use in the UK (Barrett et al. 2012; Salomone et al. 2016), low support for these children. Such inadequate provision may have long range consequences, since the coexistence of core ASD symptoms and impairments in the everyday activities that are key for an individual to function at home and fit in the community may not only profoundly affect the functioning of children, including cognitively able individuals, but also, in turn, have knock-on effects on parental wellbeing (Green and Carter 2014; Salomone et al. 2017). With respect to the HR-non-ASD group, while they had lower overall adaptive behaviour skills than LR children (and significantly better than HR-ASD children), we did not find evidence of specific relative impairments by subdomain, and mean scores were always in the average range. Our findings confirm the early emerging characteristics of lower levels of developmental and adaptive functioning related to the BAP in HR-non-ASD siblings compared to LR controls, previously reported at 36 months (Messinger et al. 2013; Charman et al. 2017).

There are a number of strengths to the present study, including the length of the follow-up and the rigorous clinical characterization of participants using measures that demonstrate internal consistency, convergent, and divergent validity in children with ASD (for the Mullen scales: Bishop et al. 2011; Swineford et al. 2015; for the WASI: Minshew et al. 2005; for the VABS: de Bildt et al. 2005; Perry and Factor 1989). Nevertheless, the results should be interpreted in the context of several limitations. Due to the wide age span, it was not possible to use a single measure of cognitive skills and the Mullen NVIQ and VIQ scores used in the analysis were obtained by averaging subtests and transforming T scores into deviation IQ scores. The imbalance in income and educational level between groups may have partly contributed to group differences in cognitive skills, particularly the LR/HR-non-ASD advantage. The HR group was also mostly comprised of cognitively able individuals: at the 84-month visit, two HR-non-ASD children had IQ in the borderline range (WASI FSIQ < 85), two HR-ASD children had intellectual disability (WASI FSIQ < 70) and all other participants displayed IQ in the average or above average range. This profile of our sample may prevent the generalizability of the findings with respect to the developmental *levels* reported, particularly for cognitive skills. Nonetheless, it may be arguable that, given the expected low correlation of adaptive and cognitive skills in the typical population (Sparrow et al. 2005), the differences among developmental *trajectories* in adaptive behaviour skills found may still hold their validity in the general population, and that findings relative to the HR-ASD children (a progressive decrease over time) may even be amplified in lower ability. Developmental trajectories of cognitive skills in a control group with average IQ should be tested in future to confirm the finding of a higher IQ in LR than in HR-non-ASD in a more representative sample.

Lastly, there was attrition from the 3-year visit to the 7-year follow-up (74% of LR children and 78% of HR children attended the 7-year visit), which may have limited the validity and generalizability of findings, given that the ASD outcome group at the 7-year visit was used as the between-subjects factor in the analysis. However, this possible limitation on the validity is mitigated by the fact that there was no systematic evidence of differences in retention based on autism severity, developmental level, adaptive behaviour or family income at the 3-year visit.

### Conclusions

This study identified distinct patterns of group differences over time for cognitive and adaptive behaviour skills in a sample of both high- and low- familial risk siblings followed prospectively from 7 months to 7 years. HR siblings, both those who were diagnosed with ASD at age 7 (HR-ASD) and those who did not (HR-non-ASD), displayed consistently lower cognitive skills over time than those of LR children. Conversely, we found a different pattern for adaptive behaviour, with HR-ASD children showing increasing difficulties over time compared to LR children, particularly in the socialization and daily living skills domains, whereas the HR-non-ASD children showed no such difficulties.

### Implications

The pattern of change in exhibited difficulties found highlights the importance of assessing developmental profiles of high-risk siblings beyond the presence of core ASD symptoms. Factors that may be related to poor adaptive skills outcomes in mid-childhood and beyond, such as atypical motor development (Travers et al. 2017), should be specifically investigated within longitudinal, familial high-risk design studies to understand possible causal mechanisms during the first years of life. Finally, analysis from larger, pooled samples, should examine whether the development of both cognitive and adaptive behaviour differs in males and females at high familial risk of ASD, as it is possible that current diagnostic procedures may underdiagnose females with ASD (Ratto et al. 2017).

With regard to the implications for clinical practice, it is worth noting that estimates of ID in the ASD population have been progressively lowering over the last decades, shifting from the majority (DeMyer et al. 1974) to a third of individuals with ASD, with a further 24% of individuals now estimated in the borderline ID range, and 44% in the average or above average range (Christensen et al. 2016). At the same time, longitudinal studies still indicate that adult outcomes are generally poor for people with ASD and suggest that a higher IQ may be a necessary but not sufficient condition for positive outcomes in areas of functioning such as relationships, employment and independent living (Howlin et al. 2013). While undoubtedly cognitive skills should be part of the core components of psycho-educational interventions for ASD, just as single unitary theoretical accounts are unlikely to wholly explain cognitive processes (Charman et al. 2011), no single cognitive component can fully predict positive outcomes. Conversely, adaptive skills, particularly in the DLS domain, are explicit, concrete and teachable competences that do not necessarily require social communication skills yet are predictive of caregivers’ wellbeing in childhood and essential requirements to live independently in adulthood. Therefore, given the heterogeneity of developmental trajectories in ASD with respect to adaptive behaviour functioning and its complex association with both core symptom severity (Szatmari et al. 2015) and cognition (Duncan and Bishop 2015), this study supports the notion that adaptive skills should be consistently targeted within comprehensive, cross-domain interventions.
